# Researches and Observations on Pelvic Hæmatocele

**Published:** 1862-06

**Authors:** 


Researches and Observations on Pelvic Hæmatocele. By J. Bryne, M.D., M.R.C.S.E., Resident Fellow of the New York Academy of Medicine, &c. New York: William Wood, 61 Walker Street. 1862.

This is a well written monograph of 44 pages, in which an obscure and important subject is ably and judiciously considered. The author defines Pelvic Hæmatocele thus:—

"The tumor to which the term hæmatocele, or hæmatoma, has been correctly applied, may be defined *an extravasation of blood into or beneath the pelvic peritonæum;* and on account of the space in which this form of tumor has been, for the most part, noticed, it has generally been described as 'recto-uterine,' 'retro-uterine,' or 'peri-uterine.' *But, as the extravasated fluid*

*does not invariably select either of the locations thus indicated*, the more correct, and at the same time comprehensive term, of *Pelvic hæmatocele* should, I think, be used, as it includes every form of this affection, whether intra or sub-peritonæal, diffused, or encysted."

In relation to the time when such tumors are most apt to occur, he says:—

"THE PERIOD OF LIFE at which hæmatocele most frequently occurs, is between 25 and 35 years, 'when the sexual system is in its greatest vigor,' and the pelvic organs are consequently more prone to congestions. For similar reasons, persons of a sanguineous temperament, or given to plethora, are most frequently the subjects of it.* It has also been generally noticed that the period of invasion is immediately before, during, or soon after the catamenial flow."

The causes and symptoms are enumerated as follows:—

"Such decided statements as these, as well as the opinions of many other high and equally reliable authorities, being entirely in accordance with my own experience and observation, I feel warranted in considering (1st), inflammation of the uterine appendages and its consequences, oftentimes the primary, and by far the most frequent among the *predisposing causes* of pelvic hæmatocele. (2d) Habitual constipation of the bowels, and morbid growths interfering with the free return of venous blood, and thereby producing a varicose condition of the vessels. (3d.) A hæmorrhagic diathesis from a disordered state of the blood. (4th.) Tubular, uterine, or vaginal occlusion, obstructing the normal secretion or giving rise to regurgitation through the Fallopian tubes. The immediate or *exciting causes* may be, (1st), sudden suppression of the menstrual, or a hæmorrhoidal discharge; (2d), tenesmus or violent muscular exertion; (3d), injuries by a fall or otherwise, and (4th), excessive coitus, and mental emotions tending to active congestion of the internal organs of generation.

"Still another cause remains to be mentioned which might, with propriety, be classed both as predisposing and exciting, namely, *extra-uterine pregnancy*."

"When the hæmorrhage takes place from the under surface of the ovary, or within the folds of the broad ligament, the patient, having previously suffered more than usual from pain in one or other iliac region, will complain suddenly of severe cramp in the lower portion of the bowels, accompanied or soon followed

* Voisin.

by tenesmus, and weight referred to the loins and sacrum; there may be painful and difficult micturition, and if the quantity of blood poured out be great, faintness, and even complete syncope may now take place; the skin assumes a pale or sometimes anæmic hue; the extremities become cold, the countenance anxious, pulse small and frequent, and the abdomen tympanitic, and very sensitive to pressure, particularly over the seat of the rupture. At this stage of the case, a vaginal examination will rarely fail to detect a tolerably firm and irregular tumor, somewhat painful to the touch, and situated directly behind, or to either side of the uterus. Before proceding further, and in order to illustrate more clearly the symptoms in detail, I will now relate the case referred to in the beginning of this paper."

On the important question of differential diagnosis the author gives the following brief summary:—

"That the diagnosis of pelvic hæmatocele must be, in many cases, extremely difficult, it is only necessary to refer to the unfortunate case reported by M. Malgaine, in 1850, where that gentleman proceded to enucleate what he, and no doubt some of his colleagues, pronounced 'fibrous tumor of the posterior wall of the uterus.' After making some free incisions, he discovered his mistake, but too late, for the patient died. And referring to this part of the subject, Nelaton says, 'the first case which I saw, I pronounced encephaloid disease, and incurable;' and of the second he adds, 'I reflected a long time, and concluded at last to introduce a trocar: if *cancer*, it could do no harm, and moreover, I had an idea that it might prove to be abscess.'

"If, however, a careful inquiry be made into the previous history of the case, I cannot perceive how it is possible to make such grave mistakes; because, malignant growths, at least, and hæmatocle, present so few symptoms in common, that unless in a very anomalous case of the latter, which might possibly exhibit some extraordinary features and complications, such an error of judgment would seem to argue great remissness on the part of the professional attendant

"The *principal* diseases with which it might be confounded, are, *pelvic abscess*, *retroversion of the uterus*, *dislocated ovarian cysts or fibrous tumors*, *and extra-uterine fœtation.* With regard to PURULENT COLLECTIONS, it might, with propriety, be hinted, that the constitutional disturbance attending this process is generally so much greater, that this one fact alone might be sufficient to settle the question, and in most cases it undoubtedly would be; but in the one which I have related, such a distinction would avail but little, since there were local as well as constitutional evidences in abundance to denote any amount of such trouble. The distinctive sign, therefore, to be most relied upon in all cases of suspected abscess, is the almost *instantaneous formation of the tumor* in hæmatocele, because, whether it be within the peritonæl cul-de-sac, or under it, and in the former, even before the inflamatory process has completed the upper boundary of the cyst, a vaginal examination will generally detect the swelling.

Retroversion at first sight might be mistaken for hæmatocele or vice versa, but the position, or rather direction of the os and cervix, and other features diagnostic of this displacement, render it hardly possible to entertain doubts on this point after mature reflection: should some peculiarities be present, however, tending to excite suspicions one way or other, the introduction of Simpson's sound will be the most unequivocal means of arriving at a correct inference.*

"Ovarian Cysts are less likely to be mistaken for hæmatocele than fibrous tumors, because, though their sudden displacement into the retro-uterine region might give rise to symtoms very analogous, yet in the former, the swelling will be more isolated and to a certain extent easily defined, movable, and situated pretty high up in the vagina: the uterus will also admit of being moved independently of the tumor, especially if the sound be used; and lastly, there will be no sudden anæmia.

"Fibrous Tumors, in the posterior uterine wall, being intimately connected with this organ, might, if examined without reference to the previous history of the case, be mistaken for hæmatocele, but with ordinary diligence in our investigation, such an error would seldom if ever occur.

"Extra-uterine Pregnancy in some of its characters, might, I imagine, resemble retro-uterine hæmatocele, but the mildness of the symtoms, some of them perhaps being those referable to ordinary pregnancy, the slowness of their developement, and the detached and movable nature of the tumor, ought to distinguish it from an *encysted* hæmatocele at least.

"As to the rupture of the fœtal envelope and discharge of its contents into the retro-uterine cul-de-cac, I will not undertake to say in what respect it would be likely to differ from intra-peritonæal extravasion; because I cannot conceive how it would

* Voisin relates a case translated from Mikschik's "Studies on the Pathology of the Ovaries," in which a hæmatocle was mistaken for "retroversion of a gravid uterus at 5m."—though the tumor was perfectly immovable, and a catheter had been introduced into the cavity of the uterus!

be possible to separate the symtoms of the one from the other, with any degree of certanity, in almost any given case.

"Lest it might be supposed, from the brief—not to say incomplete—manner in which I have referred to the various diseases and conditions of the pelvic viscera with which hæmatocele might be confounded, that I do not sufficiently appreciate the many and great difficulties attending the diagnosis of this affection, I cannot dismiss the subject without recording my firm conviction, that in very many cases an accurate idea of the nature of these tumors will be next to impossible until the trocar shall have penetrated their walls: and should any one feel so self sufficient as to believe otherwise, I would refer him to the pages of Voisin, where he will find it recorded of Nelaton, that even so late as 1857 that justly celebrated surgeon mistook a case of post-uterine abcess for hæmatocele; of MM. Robert and Huguier, who pronounced a case of extra-uterine pregnancy an hæmatocele; of Dupis, who relates an instance of serous cyst having been taken for hæmatocele; and of Prof. Stoltz, whose error in diagnosis was similar to that of M. Malgaigne."

The treatment of pelvic hæmatocele must depend somewhat on the size and location of the tumor, and the inflamatory symptoms accompanying it. If the latter are severe they must be relieved by the means usually relied on for combating local inflammation. If the quantity of accumulated blood is small, it may eventually disappear by absorption. Generally, however, the tumor will not disappear until a puncture is made sufficient to discharge its contents. The extracts we have made, will show the author's style and manner of treating his cubject; but we would advise all to read the essay for themselves.

